# Immune-complex glomerulonephritis in cats: a retrospective study based on clinico-pathological data, histopathology and ultrastructural features

**DOI:** 10.1186/s12917-019-2046-y

**Published:** 2019-08-20

**Authors:** Francesco Rossi, Luca Aresu, Valeria Martini, Davide Trez, Rossella Zanetti, Luigi Michele Coppola, Filippo Ferri, Eric Zini

**Affiliations:** 1 Istituto Veterinario di Novara, Strada Provinciale 9, 28060 Granozzo con Monticello (NO), Novara, Italy; 2 Department of Veterinary Science, University of Turin, Largo Braccini 2, 10095 Grugliasco (TO), Italy; 3 Department of Veterinary Medicine, University of Milan, via dell’Università, 26900 Lodi, Italy; 4 Department of Comparative Biomedicine and Food Science, University of Padua, Viale dell’Universita 16, 35020 Agripolis Legnaro (PD), Legnaro, Italy; 5 Department of Animal Medicine, Production and Health, University of Padua, Viale dell’Universita 16, 35020 Agripolis Legnaro (PD), Legnaro, Italy; 6 Clinic for Small Animal Internal Medicine, Vetsuisse Faculty, University of Zurich, Winterthurerstrasse 260, 8057 Zurich, Switzerland

**Keywords:** Feline, Kidney, Proteinuria, Transmission electron microscopy, Immune-deposits

## Abstract

**Background:**

Chronic kidney disease (CKD) has typically a non-immune mediated origin in cats and immune-complex glomerulonephritis (ICGN) is scarcely described. Aims of this study were to characterize ICGN by light and electron microscopy and identify associations with clinico-pathological findings. In addition, comparisons between cats with ICGN and non immune-complex glomerulonephritis (non-ICGN) were performed.

Renal samples examined between 2010 and 2019 were considered if both light and electron microscopy were performed. Signalment, feline immunodeficiency virus (FIV) and leukemia virus (FeLV) status, serum creatinine concentration, urine protein-to-creatinine (UPC) ratio, systolic blood pressure (SBP) and International Renal Interest Society (IRIS) stage were retrieved and used for comparisons.

**Results:**

Sixty-eight client-owned cats were included. Thirty-seven cats (54.4%) had ICGN and 31 (45.6%) non-ICGN. Eighteen (48.6%) with ICGN had membranous glomerulonephropathy (MGN), 14 (37.8%) membranoproliferative glomerulonephritis (MPGN), and 5 (13.5%) mesangioproliferative glomerulonephritis (MeGN). Clinico-pathological data were not associated with any type of ICGN. Among cats with non-ICGN, 11 (35.5%) had end-stage CKD, 9 (29%) focal segmental glomerulosclerosis, 6 (19.4%) global and multifocal mesangiosclerosis, 2 (6.5%) glomerular atrophy, 2 (6.5%) renal dysplasia and 1 (3.1%) amyloidosis. Eight (25.8%) cats with non-ICGN had chronic interstitial nephritis (CIN) grade 1, 13 (41.9%) grade 2 and 10 (32.3%) grade 3; creatinine and UPC ratio increased with CIN grades (*p* = 0.001, *p* < 0.001). Cats with ICGN were more frequently FIV or FeLV-infected (OR:11.4; 95%CI:1.4–94.4; *p* = 0.024), had higher UPC ratio (OR:6.8; 95%CI:2.5–18.2; p < 0.001) and were younger (OR:0.9; 95%CI:0.7–1.0; *p* = 0.042) than cats with non-ICGN.

**Conclusions:**

MGN and MPGN were the most common morphological diagnoses of ICGN in cats. Unfortunately, none of the investigated findings differentiated ICGN morphological diagnoses. Serum creatinine concentration and UPC ratio were directly associated with grades of CIN (p = 0.001 and p < 0.001, respectively), confirming previous literature. More ICGN than non-ICGN was observed in cats with retroviral infections, younger cats and higher UPC ratio.

## Background

Recently, the World Small Animal Veterinary Association Renal Standardization Study Group (WSAVA-RSSG) has provided criteria to diagnose glomerular diseases in dogs based on light microscopy (LM) and transmission electron microscopy (TEM), as well as immunofluorescence [1]. According to the WSAVA-RSSG, two broad diagnostic categories have been identified, namely immune-complex glomerulonephritis (ICGN) and non-immune-complex glomerulonephritis (non-ICGN) [1]. Because specific morphological criteria for glomerular diseases have not been reported in cats, the above classification scheme is commonly adopted in the feline species. However, to date only few reports of ICGN in cats have been published and membranous glomerulonephropathy (MGN) was reported most frequently [2–6], often associated with feline leukemia virus (FeLV) infection [4, 6–8]. ICGN has also been described in feline immunodeficiency virus (FIV) positive cats and mesangioproliferative glomerulonephritis (MeGN) was the most common lesion [9]. Further, MeGN has been reported in a cat with cyanotic congenital heart disease, as observed in humans who developed transient proteinuria and edema [10, 11]. In addition, feline coronavirus leading to feline infectious peritonitis (FIP) has been associated with various types of ICGN, including MGN, MeGN and membranoproliferative glomerulonephritis (MPGN) [12]. Finally, MPGNs resembling human type I and III have been described in two cats [3, 13], as well as proliferative, necrotizing and crescentic ICGN in a cat [14].

Chronic kidney disease (CKD) secondary to non-ICGN is much more frequent than ICGN, with the majority of cats having non-specific renal lesions [15, 16]. It is usually documented in aged cats and chronic interstitial nephritis (CIN) associated with secondary glomerular involvement is the predominant morphologic diagnosis [15–17]. However, no studies have detailed the ultrastructural lesions in the glomeruli of these cats, thereby reducing diagnostic specificity and sensibility. Based on the severity of interstitial fibrosis and inflammatory infiltration, CIN is currently histologically classified according to a scale that ranges from 0 to 3, in ascending order of severity [17]. The amount of fibrosis represents the lesion that best correlates with the severity of azotemia, hyperphosphatemia and anemia, while glomerular hypertrophy correlates best with the severity of proteinuria [17].

Large cohort studies describing ICGN in cats are still lacking because cases with primary glomerular lesions are seldom encountered and the use of renal biopsy is yet very limited in clinical practice. Therefore, the aims of this study were first to characterize morphological diagnosis of ICGN with LM and TEM in a large group of cats and to identify associations with clinical and laboratory findings. Secondly, a group of non-ICGN cats were used for comparison to highlight the need of histopathology and electron microscopy to obtain a final diagnosis.

## Results

### Cats with ICGN and clinical features associated with morphological diagnoses

Of 37 cats diagnosed with ICGN, breed was known for 36; 34 (94.4%) were DSH and 2 (5.6%) purebred, including 1 each of Norwegian Forest and Abyssinian.

Concerning FIV and FeLV status, all cats had been tested and 13 (35.1%) cats were positive for retroviral infection, including 10 FIV-positive cats and 3 FeLV-positive; none was concurrently infected with both viruses. The mean serum creatinine concentration was 3.3 ± 2.3 mg/dL (median: 2.6 mg/dL; min-max: 0.6–11.1 mg/dL). Mean UPC ratio at diagnosis was 7 ± 3.2 (median: 2.6; min-max 2.4–18.6). The mean SBP value was 154 ± 25 mmHg (median: 160 mmHg; min-max 110–200 mmHg); SBP was not available in 10 cats. Five (13.5%) cats were in IRIS stage 1, 15 (40.5%) in stage 2, 11 (29.7%) in stage 3 and 6 (16.2%) in stage 4.

**Fig. 1:**
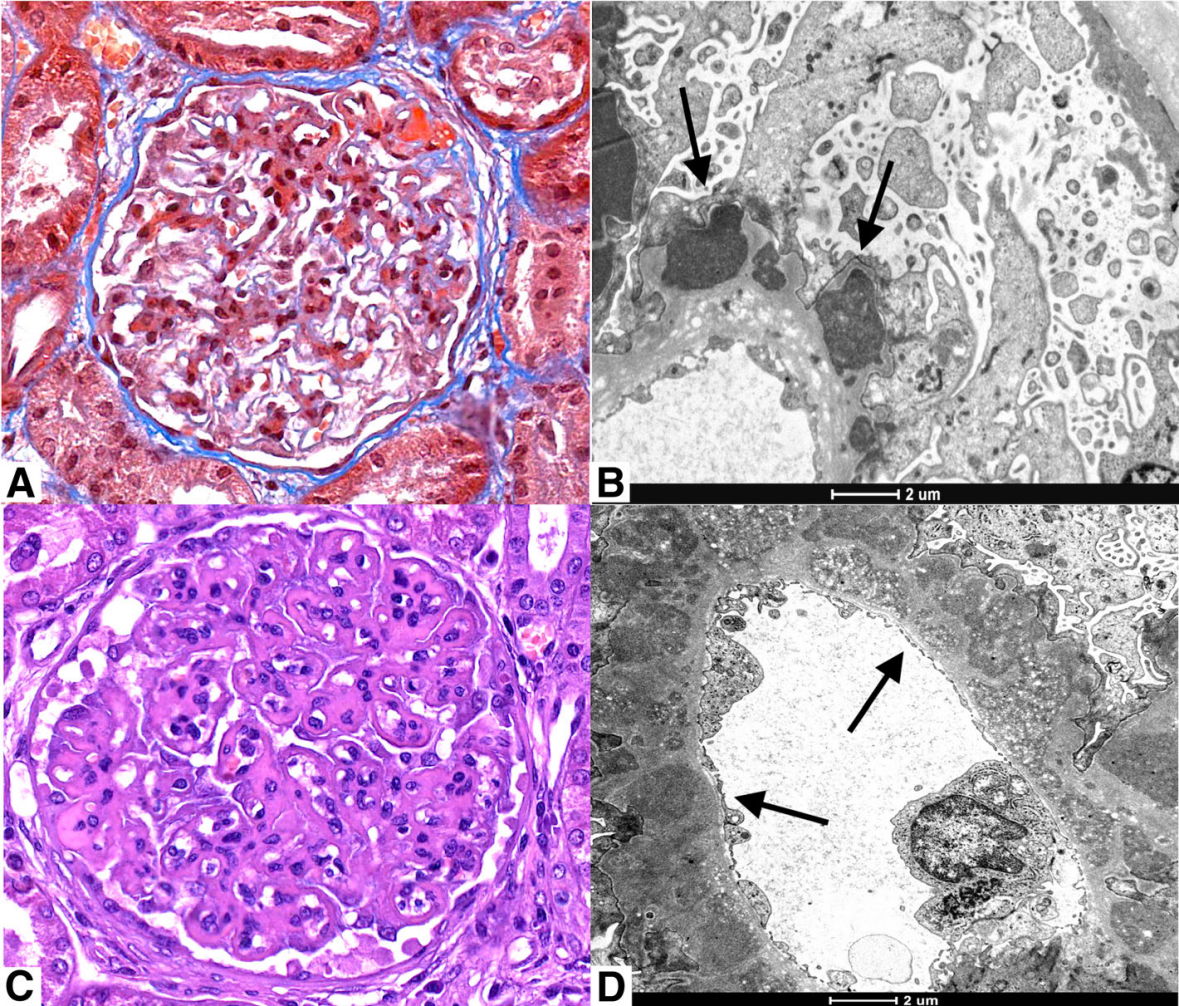
Histological and ultrastructural findings in cases of MGN (**a**, **b**) and MPGN (**c**, **d**). Masson’s Trichrome section of a glomerulus with moderate mesangial sclerosis and thickening of glomerular basement membrane associated to protein deposits (**a**). Electron microscopy shows sub-epithelial electron dense deposits in the GBM (arrows) (**b**). PAS section of a glomerulus shows remodeling of the glomerular basement membrane, mesangial interposition and mesangial matrix increase (**c**). Electron microscopy reveals immune complexes in the basement membrane associated with an increase of thickening (arrows) (**d**)

Morphological diagnoses of cats affected by ICGN were MGN (Fig. 1A, B) in 18 (48.6%), MPGN (Fig. 1C, D) in 14 (37.8%), and MeGN in 5 (13.5%). Of the 10 FIV-positive cats, 5 had MGN, 4 MPGN, and 1 MeGN; of the 3 FeLV-positive cats, 1 each had MGN, MPGN and MeGN.

Breed, sex, age, FIV and FeLV status, UPC ratio, serum creatinine concentration, SBP and IRIS stage in cats with different ICGN subtypes are shown in Table 1.

**Table 1:** Breed, sex, age, FIV and FeLV status, serum creatinine concentration, UPC ratio, SBP and IRIS stage in cats with different morphological types of ICGN

Variable	ICGN subtype # of cats (%)		
	MGN	MPGN	MeGN
Breed: DSH vs. purebred			
absolute number	17 vs. 0	13 vs. 1	4 vs. 1
(%)	(100 vs. 0)	(92.9 vs. 7.1)	(80 vs. 20)
Sex: SF vs. NM			
absolute value	7 vs. 11	6 vs. 8	3 vs. 2
(%)	(38.9 vs. 61.1)	(42.9 vs. 57.1)	(60 vs. 40)
Age (years)			
mean ± sd	8.7 ± 2.8	9.1 ± 3.6	11 ± 4.2
(median; range)	(9; 4–14)	(9; 4–15)	(12; 4–15)
FIV status: + vs. -			
absolute value	5 vs. 13	4 vs. 10	1 vs. 4
(%)	(27.8 vs. 72.2)	(28.6 vs. 71.4)	(20 vs. 80)
FeLV status: + vs. -			
absolute value	1 vs. 17	1 vs. 13	1 vs. 4
(%)	(5.3 vs. 94.4)	(7.1 vs. 92.9)	(20 vs. 80)
Creatinine (mg/dL)			
mean ± sd	3 ± 2.3	3.7 ± 2.6	3.3 ± 1.7
(median; range)	(2.3; 1.2–11.1)	(2.9; 1.1–9.5)	(3.4; 0.6–5.2)
UPC ratio			
mean ± sd	7.6 ± 4	6.4 ± 1.9	7 ± 3.3
(median; range)	(6.6; 2.4–18.6)	(6.5; 3.9–10.5)	(5.4; 3.4–11.4)
SBP (mmHg)			
mean ± sd	155 ± 30	156 ± 19	147 ± 32
(median; range)	(155; 120–200)	(160; 120–190)	(160; 110–170)
IRIS stage: 1 vs 2 vs 3 vs 4	3 vs 9 vs 4 vs 2	1 vs 6 vs 4 vs 3	1 vs 0 vs 3 vs 1
absolute value	(16.7 vs 50.0 vs	(7.1 vs 42.9 vs 28.6	(20 vs 0 vs 60 vs
(%)	22.2 vs 11.1)	vs 21.4)	20)

ICGN, immune-complex glomerulonephritis; MGN, membranous glomerulonephropathy; MPGN, membranoproliferative glomerulonephritis; MeGN, mesangioproliferative glomerulonephritis; DSH, domestic short-haired; SF, spayed female; NM, neutered male; FIV, feline immunodeficiency virus; FeLV, feline leukemia virus; sd, standard deviation; UPC, urine protein-to-creatinine; SBP, systolic blood pressure; IRIS, International Renal Interest Society

None of the investigated variables were significantly associated with the ICGN diagnoses.

### Cats with non-ICGN and clinical features associated with CIN grades

Overall, 31 cats had non-ICGN renal disease, including 11 (35.5%) end-stage CKD, 9 (29%) focal segmental glomerulosclerosis, 6 (19.4%) global and multifocal glomerulosclerosis, 2 each of glomerular atrophy (6.5%) and renal dysplasia (6.5%), and 1 amyloidosis (3.1%).

Twenty-five (80.6%) were DSH and 6 (19.4%) were purebred, including 2 Persians, 1 each of Siamese, Chartreux, Maine Coon and Norwegian Forest breeds. Twenty-one cats were tested for FIV and FeLV infection and none was positive.

Serum creatinine concentration and UPC ratio were available in all cats, and the mean values were 3.6 ± 2.3 mg/dL (median: 3.2 mg/dL; min-max: 0.6–11.0 mg/dL) and 2.6 ± 0.9 (median: 2.6; min-max 1.0–4.5), respectively. SBP was known for 18 cats and the mean value was 154 ± 30 mmHg (median: 150 mmHg; min-max 110–200 mmHg). Seven (22.6%) cats were in IRIS stage 1, 6 (19.4%) in stage 2, 11 (35.5%) in stage 3, and 7 (22.6%) in stage 4.

Eight (25.8%) cats diagnosed with non-ICGN had CIN grade 1, 13 (41.9%) had grade 2, and 10 (32.3%) had grade 3. Clinico-pathological data for each CIN grade are reported in Table 2. There was no difference in sex and breed distribution among CIN grades, as well as for mean age and SBP. Conversely, serum creatinine concentration and UPC ratio significantly varied with CIN grades (*p* = 0.001 and *p* < 0.001, respectively). In particular, cats with CIN grade 1 had significantly lower serum creatinine concentration and UPC ratio and compared to those with CIN grade 2 (*p* = 0.010 and p < 0.001, respectively) and to those with CIN grade 3 (*p* < 0.001 for both analyses). Differences in serum creatinine concentration and UPC ratio between CIN grade 2 and grade 3 were not statistically significant based on post-hoc analyses.

**Table 2:** Breed, sex, age, serum creatinine concentration, UPC ratio, SBP and IRIS stage in cats for each CIN grade

Variable	CIN grade # of cats (%)		
	1	2	3
Breed: DSH vs. purebred			
absolute number	6 vs. 2	10 vs. 3	9 vs. 1
(%)	(75 vs. 25)	(76.9 vs. 23.1)	(90 vs. 10)
Sex: SF vs. NM			
absolute value	4 vs. 4	8 vs. 5	7 vs. 3
(%)	(50 vs. 50)	(61.5 vs. 38.5)	(70 vs. 30)
Age (years)			
mean ± sd	11.6 ± 3.8	9.8 ± 2.6	11.8 ± 3.6
(median; range)	(12.5; 6–17)	(10; 5–14)	(13; 4–15)
Creatinine (mg/dL)			
mean ± sd	1.5 ± 0.8	4 ± 2.9	4.8 ± 0.9
(median; range)	(1.3; 0.6–3.2)	(3.1; 1–11)	(4.6; 3.4–6.2)
UPC ratio			
mean ± sd	1.5 ± 0.5	2.7 ± 0.7	3.3 ± 0.6
(median; range)	(1.5; 1–2.3)	(2.6; 1.7–4.3)	(3.1; 2.6–4.5)
SBP (mmHg)			
mean ± sd	145 ± 7	149 ± 28	164 ± 37
(median; range)	(145; 140–150)	(140; 120–190)	(180; 110–200)
IRIS stage: 1 vs 2 vs 3 vs 4	5 vs 2 vs 1 vs 0	2 vs 4 vs 3 vs 4	0 vs 0 vs 7 vs 3
absolute value	(62.5 vs 25.0 vs	(15.4 vs 30.8 vs	(0 vs 0 vs 70 vs 30)
(%)	12.5 vs 0.0)	23.1 vs 30.8)	

CIN, chronic interstitial nephritis; DSH, domestic short-haired; SF, spayed female; NM, neutered male; sd, standard deviation; UPC, urine protein-to-creatinine; SBP, systolic blood pressure; IRIS, International Renal Interest Society

The proportion of cats classified in the different IRIS stages significantly varied with CIN grades (*p* = 0.005). In particular, none of the cats with CIN grade 1 was in IRIS stage 4, and none of the cats with CIN grade 3 was in IRIS stage 1 or 2. Cats with CIN grade 2 were distributed homogeneously among the four IRIS stages.

### Diagnosis of ICGN or non-ICGN based on clinical and laboratory data

According to logistic regression analysis, FIV or FeLV infection, age and UPC ratio were significantly associated with the likelihood of diagnosing ICGN rather than non-ICGN (*p* = 0.024, *p* = 0.042 and *p* < 0.001, respectively). In particular, the likelihood of diagnosing ICGN increased by 11.4-fold for FIV or FeLV infected cates (OR: 11.4; 95% CI: 1.4–94.4) and by 6.8-fold for each 1-unit (1.0) increase of UPC ratio (OR: 6.8; 95% CI: 2.5–18.2). Conversely, the likelihood of diagnosing non-ICGN increased by 1.2-fold for each increase of year of age (OR: 1.2; 95% CI: 1.0–1.4).

With regard to the UPC ratio, the best cut-off to differentiate between cats with ICGN and those with non-ICGN was 3.8, with a 91.9% sensitivity and 93.5% specificity: the probability of diagnosing ICGN was significantly higher in cats with UPC > 3.8 (*p* < 0.001; OR 164.3; 95% CI 25.7–1052.0). In particular, 3 of 37 cats diagnosed with ICGN (8%) had UPC ratio < 3.8 and the remaining 34 (92%) > 3.8, while 29 of 31 cats diagnosed with non-ICGN (93.5%) had UPC ratio < 3.8 and the remaining 2 (6.5%) had UPC ratio > 3.8. The 2 cats diagnosed with non-ICGN and having UPC ratio > 3.8 had serum creatinine concentration of 5.0 and 7.8 mg/dL and severe glomerular involvement as well as CIN grade 2 and 3, respectively.

Cats diagnosed with ICGN had higher UPC ratio than non-ICGN cats with CIN grade 1, grade 2 and grade 3 (p < 0.001 for all analyses). They also had higher serum creatinine concentration than non-ICGN cats with CIN grade 1 and grade 3 (*p* = 0.005 and *p* = 0.002, respectively) and were younger than cats with CIN grade 3 (*p* = 0.036).

The proportion of cats classified in each IRIS stage did not significantly vary between ICGN and non-ICGN cats. Still, significant differences were found when comparing cats with ICGN to those with CIN grade 1 and grade 3 (*p* = 0.023 for both analyses). Compared to cats affected by ICGN, cats with CIN grade 1 were more commonly in IRIS stage 1, whereas cats with CIN grade 3 were less commonly in IRIS stages 1 and 2.

Lastly, breed and sex distribution, serum creatinine concentration and SBP were not associated with the chance of diagnosing ICGN or non-ICGN.

Results of breed, sex, age, FIV and FeLV status, UPC ratio, serum creatinine concentration, SBP and IRIS stage in cats with ICGN or non-ICGN are shown in Table 3.

**Table 3:** Breed, sex, age, FIV and FeLV status, serum creatinine concentration, UPC ratio, SBP and IRIS stage in cats with ICGN and in cats with non-ICGN, and their influence on the chance to make either diagnosis

Variable	Disease category		*p* value
	ICGN	Non-ICGN	
Breed: DSH vs. purebred			
absolute number	34 vs. 2	25 vs. 6	0.101
(%)	(94.4 vs. 5.6)	(80.6 vs. 19.4)	
Sex: SF vs. NM			
absolute value	16 vs. 21	19 vs. 12	0.140
(%)	(43.2 vs. 56.8)	(61.3 vs. 38.7)	
Age (years)			
mean ± sd	8.9 ± 3.5	10.3 ± 3.4	0.042
(median; range)	(9; 4–15)	(11; 4–17)	
FIV or FeLV status: + vs. -			
absolute value	13 vs. 24	0 vs. 21	0.024
(%)	(35.1% vs. 64.9%)	(0% vs. 100%)	
Creatinine (mg/dL)			
mean ± sd	3.5 ± 2.6	4.4 ± 2.6	0.612
(median; range)	(2.4; 0.6–11.1)	(4.3; 0.6–11.0)	
UPC ratio			
mean ± sd	7.5 ± 3.5	2.8 ± 0.9	< 0.001
(median; range)	(6.6; 2.4–18.6)	(2.9; 1.0–4.5)	
SBP (mmHg)			
mean ± sd	154 ± 25	154 ± 30	0.999
(median; range)	(160; 110–200)	(150; 110–200)	
IRIS stage: 1 vs 2 vs 3 vs 4	5 vs 15 vs 11 vs 6	7 vs 6 vs 11 vs 7	0.304
absolute value	(13.5 vs 40.5 vs 29.7	(22.6 vs 19.4 vs 35.5	
(%)	vs 61.2)	vs 22.6)	

ICGN, immune-complex glomerulonephritis; non-ICGN, non immune-complex glomerulonephritis; DSH, domestic short-haired; SF, spayed female; NM, neutered male; FIV, feline immunodeficiency virus; FeLV, feline leukemia virus; sd, standard deviation; UPC, urine protein-to-creatinine; SBP, systolic blood pressure; IRIS, International Renal Interest Society

## Discussion

To date, there are few available data on ICGN in cats [2–6]. In the current study, MGN and MPGN were the two most common types, representing approximately more than three-quarters of all ICGN cases. This differs from previous investigations where MGN was the most frequent form of ICGN in cats [2–6]. One possible explanation is that the number of FeLV-positive cats with ICGN was very low (only 3 cases) compared to former studies [4, 6–8], and this retrovirus infection has been associated most commonly with the development of MGN [7]. Of note, although only 3 cats in the present study had FeLV infection, one of the 3 had MGN, while the remaining 2 had MPGN and MeGN, suggesting that FeLV-positive cats may also be affected by other types of ICGN.

ICGN was not associated with breed, age, gender, serum creatinine concentration, UPC ratio and SBP. Therefore, unfortunately, the above clinical and laboratory findings cannot be used in daily practice to predict the type of ICGN. The lack of association between the degree of proteinuria and the type of ICGN has been also reported in dogs [1, 18].

Concerning the likelihood of diagnosing ICGN vs non-ICGN based on clinical and laboratory data, values of the UPC ratio were potentially useful to anticipate the category of renal disease in cats. In particular, higher severity of proteinuria was significantly associated with ICGN.

In this study, the UPC ratio of cats with ICGN was 7 ± 3.2 (median: 2.6; min-max 2.4–18.6). Based on the notion that animals with glomerular diseases are expected to have marked proteinuria [20, 21], a higher UPC ratio would be expected if ICGN is diagnosed rather than non-ICGN, which is in line with our findings. In particular, all cats with ICGN had UPC ratio ≥ 2, which is considered indicative of the presence of glomerular disease in persistent proteinuria [20, 21].

Of note, tubular proteinuria associated with CIN is usually low-grade and glomerular proteinuria can be of any magnitude, ranging from low-grade to substantial [22]. In the present series, the best discriminating cut-off for UPC ratio was 3.8, with a high sensitivity and specificity (both > 91%). This result confirms that cats with ICGN are more likely to present with significant proteinuria, whereas some may have lesser degrees of protein loss. However, clinicians should be aware that cats with a higher degree of proteinuria might not necessarily have ICGN, but a non-immune complex disease such as amyloidosis or focal and segmental glomerulosclerosis with high CIN grade. Therefore, any therapy with immunosuppressive drugs should be carefully considered and in light of immune deposits potentially detected at TEM.

Cats with FIV or FeLV infection were 11-times more likely to be diagnosed with ICGN than non-ICGN. A possible explanation is that retroviral infections play a role in glomerular damage by promoting deposition of immune complexes derived from viral antigens and host antibodies [6, 7]. A marked increase of circulating immune complexes has been demonstrated in FIV-positive cats compared to negative ones [23]. Although the overall number of retrovirus-infected cats was relatively low in this study, FIV was three times as common as FeLV and cats with the former disease had various types of ICGN. In previous studies, ICGN was often associated with FeLV infection [4, 6–8], while in FIV-positive cats ICGN was infrequent and only MeGN was reported in these cases [9]. Thus, the data from the current study suggest that infection with FIV may underlie ICGN in cats and may lead to different types of ICGN.

Moreover, occurrences of ICGN and non-ICGN were not associated with breed, sex, creatinine concentration and SBP values. Of note, cats with non-ICGN would be expected to have higher creatinine compared to cats with ICGN because tubulointerstitial damage is typically associated with reduced glomerular filtration rate and increased serum markers of renal function [16].

Serum creatinine concentrations and UPC ratio were significantly higher in cats with CIN grade 2 and 3 than in those with grade 1; in addition, the CIN grade significantly varied between the four IRIS stages, and none of the cats with IRIS stages 1 and 2 had CIN grade 3. These data confirm previous studies where the degree of proteinuria was associated with severity of tubular degeneration, inflammation, fibrosis and necrosis, as well as with the presence of glomerular hypertrophy, while the severity of azotemia, hyperphosphatemia and anemia were associated with the extent of fibrosis [16, 17].

Interestingly, a number of cats with non-ICGN had UPC ratios > 2. This finding can be explained by the fact that in this group of cats CIN were diagnosed along with a variety of glomerular lesions and that the latter likely represented the major contributors to protein loss.

This study has some limitations, such as its retrospective nature and the fact that data were provided by different veterinarians. Indeed, incomplete records were present and information pertaining to serum albumin concentrations or to ascites was scant. However, because missing data were documented both in ICGN and non-ICGN cats, the bias was likely reduced. Another important limitation is that blood tests and urinalysis were performed in different laboratories, with reference ranges that might have differed. In addition, the UPC ratios provided in this study were likely based on single determinations in most cats without accounting for day-to-day variability of proteinuria. However, the authors assume that the potential bias of having different laboratories and collection of single urine samples were probably evenly distributed among morphological diagnoses and disease categories, reducing its confounding effect. Another limit of this study is the lack of immunofluorescence data. Indeed, immunofluorescence as well as LM and TEM may help characterizing glomerulonephritis in cats and provide a complete evaluation of renal biopsies.

## Conclusions

MGN and MPGN were the two most common morphological diagnoses of ICGN in cats. Unfortunately, clinical and laboratory findings were not useful to differentiate among specific types of ICGN. In cats with non-ICGN, only serum creatinine concentration and UPC ratio were significantly associated with CIN grades. Cats with ICGN were more likely to be infected with FIV or FeLV, had more severe proteinuria and were younger than cats with non-ICGN. Similar to dogs, renal biopsies in cats with glomerulopathy should include both LM and TEM examination in order to achieve a final morphological diagnosis and, potentially, to tailor treatment.

## Methods

### Cats and renal samples

Tru-cut renal biopsies of cats with renal disease caused by ICGN that were submitted to the European Veterinary Renal Pathology Service (http://www.evrps.net) and Department of Veterinary Science in the University of Turin between 2010 and 2019 were retrospectively analysed. Additionally, kidney samples obtained from cats autopsied for any disease were included if a non-ICGN was identified. Cases were considered if renal samples were collected within 1 h from death and if there was an adequate amount of tissue to achieve diagnosis with both LM and TEM. Furthermore, the last available clinical and laboratory data reported in medical records were retrieved if obtained within a month prior to renal sampling. Sixty-eight cats fulfilled the inclusion criteria. Among them, 37 (54.4%) were diagnosed with ICGN and 31 (45.6%) with non-ICGN. Among cats with ICGN 21 (56.8%) were neutered males and 16 (43.2%) were spayed females. None of the cats was intact. The mean age was 9.2 ± 3.3 years (median: 9 years; min-max: 4–15 years). Of cats with non-ICGN, 19 (61.3%) were spayed female and 12 (38.7%) were neutered male. None of the cats was intact. Mean age was 10.9 ± 3.3 years (median: 11 years; min-max: 4–17 years).

With regards to kidney samples, formalin-fixed and paraffin-embedded renal tissues were stained with haematoxylin and eosin (HE), periodic acid-Schiff (PAS), Masson’s trichrome, and periodic acid-Schiff methenamine silver (PASM) or Jones Methenamine silver. Renal tissues for TEM were always fixed in glutaraldehyde and analysis was performed using standard procedures as previously described [18]. To achieve a final diagnosis of ICGN, glomerular immune-deposits had to be identified with TEM and, according to their location, ICGNs were further classified in MPGN, MGN and MeGN, following the WSAVA-RSSG classification scheme used in dogs [1].

Renal samples of cats diagnosed with non-ICGN were histologically characterized by different morphologic pattern of glomerular diseases, including amyloid deposition, podocytes damage, global and multifocal glomerulosclerosis and renal maldevelopment. Tubulointerstitial damage was a common feature [16, 17]. The TEM was always available and used to confirm the absence of glomerular immune-deposits and to characterize glomerular lesions. Furthermore, in these cats CIN was scored on a scale from 1 to 3, as follows: 1 = mild or scattered multifocal areas of fibrosis and inflammation affecting < 5% of the section, 2 = moderate fibrosis or inflammation affecting 25 to 50% of the section, 3 = diffuse or coalescing fibrosis or inflammation [17].

### Statistical analysis

The following variables were included in statistical analyses: age (years), sex (spayed female or neutered male), breed [domestic short-haired (DSH) or purebred], FIV or FeLV infection (positive or negative), urine protein-to-creatinine (UPC) ratio, serum creatinine concentration (mg/dL), International Renal Interest Society (IRIS) stage (1 to 4) [19], and systolic blood pressure (SBP; mmHg). Continuous data are shown as mean and standard deviation, median and range, whereas categorical data are shown as percentages.

For continuous variables, normal distribution of data was tested with a Shapiro-Wilk test. Thereafter, possible differences among ICGN subtypes and CIN grades of non-ICGN were investigated via Kruskal-Wallis or Analysis of Variance (ANOVA) tests. When a significant variation occurred, post-hoc analyses were performed using Mann-Whitney, Bonferroni or Dunnett test, based on data distribution and homoscedasticity assessment. Mann-Whitney and Student t-test were used to investigate possible differences between cats with ICGN and each CIN grade, respectively.

Differences for categorical variables among ICGN subtypes, among CIN grades, and between cats with ICGN and each CIN grade, were investigated by means of contingency tables and Pearson chi-square test. Fisher’s exact test was applied to 2 × 2 contingency tables when appropriate.

To detect whether any of the investigated variables influenced the likelihood of diagnosing ICGN or non-ICGN renal disease, binomial logistic regression was performed. For laboratory data giving significant result, receiver operating characteristic (ROC) curves were drawn to identify the cutoff most suitable to discriminate between ICGN and non-ICGN cats. Different groups were created based on the selected cutoff, and logistic regression was performed to assess possible association between the newly constituted groups and the likelihood of diagnosing ICGN or non-ICGN. Odd ratios (OR) and their respective 95% confidence intervals (CI) were also calculated for variables yielding significant results.

Significance was set at *p* ≤ 0.05 for all tests. This was lowered to *p* ≤ 0.017 for Mann-Whitney test whereby it was used for post-hoc analyses, in order to reduce the family-wise error rate in multiple comparisons. Analyses were performed using a commercial software package (SPSS v20.0 for Windows, Chicago, IL).

## Data Availability

The datasets used and/or analysed during the current study are available from the corresponding author on reasonable request.

## Abbreviations

CIN: Chronic interstitial nephritis
CKD: Chronic kidney disease
DSH: Domestic short-haired
FeLV: Feline leukemia virus
FIV: Feline immunodeficiency virus
ICGN: Immune-complex glomerulonephritis
IRIS: International Renal Interest Society
LM: Light microscopy
MeGN: Mesangioproliferative glomerulonephritis
MGN: Membranous glomerulonephropathy
MPGN: Membranoproliferative glomerulonephritis
Non-ICGN: Non immune-complex glomerulonephritis
SBP: Systolic blood pressure
TEM: Transmission electron microscopy
UPC: Urine protein-to-creatinine
WSAVA-RSSG: World Small Animal Veterinary Association Renal Standardization Study Group
